# Relating Education, Brain Structure, and Cognition: The Role of Cardiovascular Disease Risk Factors

**DOI:** 10.1155/2014/271487

**Published:** 2014-08-12

**Authors:** Moyra E. Mortby, Richard Burns, Andrew L. Janke, Perminder S. Sachdev, Kaarin J. Anstey, Nicolas Cherbuin

**Affiliations:** ^1^Centre for Research on Ageing, Health and Wellbeing, The Australian National University, 62A Eggleston Road, Canberra, ACT 0200, Australia; ^2^Centre for Advanced Imaging, The University of Queensland, 57 Research Road, Brisbane, QLD 4072, Australia; ^3^Neuropsychiatric Institute, Euroa Centre, Prince of Wales Hospital, University of New South Wales, Sydney, NSW 2031, Australia

## Abstract

The protective effect of education on cognitive and brain health is well established. While the direct effects of individual cardiovascular disease (CVD) risk factors (i.e., hypertension, smoking, diabetes, and obesity) on cerebral structure have been investigated, little is understood about the possible interaction between the protective effect of education and the deleterious effects of CVD risk factors in predicting brain ageing and cognition. Using data from the PATH Through Life study (*N* = 266), we investigated the protective effect of education on cerebral structure and function and tested a possible mediating role of CVD risk factors. Higher education was associated with larger regional grey/white matter volumes in the prefrontal cortex in men only. The association between education and cognition was mediated by brain volumes but only for grey matter and only in relation to information processing speed. CVD risk factors did not mediate the association between regional volumes and cognition. This study provides additional evidence in support for a protective effect of education on cerebral structures and cognition. However, it does not provide support for a mediating role of CVD risk factors in these associations.

## 1. Introduction

Numerous epidemiological, imaging, neuropsychological, and pathological studies have highlighted the protective effects of higher educational attainment (for a review see [1]), linking it to more successful ageing [2], better cognitive test performance [3–5], slower cognitive and functional decline [6–12], lower incidence rates of dementia [13–15], and larger regional brain volumes [16–18]. Traditionally, the observed protective effects of education have been explained within the theoretical context of the cognitive reserve (CR) hypothesis [19]. Specifically, the concept of CR is used to explain the capacity of more highly educated individuals to tolerate a greater level of age- and disease-related pathology without obvious clinical symptom manifestation by protecting or providing resilience against neurodegenerative pathologies [8, 9, 19–24]. Education, amongst other factors (e.g., occupation, intellectual functioning/IQ, stimulating cognitive, and social and physical activities), is considered a determinant and index of CR [25, 26].

The CR hypothesis is commonly used to provide an explanation for the repeated clinical observation showing large discrepancies between measured brain pathology (e.g., cerebral amyloidosis) and cognitive decline [27–30], as well as prolonged durations of relatively preserved cognitive function in highly educated individuals with Alzheimer's disease [8, 9]. Several neuroimaging studies have investigated the brain substrates associated with education-related CR and provided support for the hypothesis by demonstrating that education has a protective effect on brain ageing in healthy elderly and those suffering from mild cognitive impairment and Alzheimer's disease (e.g., [16, 31, 32]). Specifically, these studies have linked higher levels of education with greater grey matter volumes and cortical thickness in the temporal, temporoparietal, and orbitofrontal cortices and greater white matter volumes in the inferior frontal areas, as well as increased fibre connectivity in white matter structures connecting the temporoparietal and orbitofrontal lobes [16–18, 33], decreased fibre integrity in the hippocampus [34], and reduced cortical atrophy [35]. In summary, these findings highlight the protective effect of early-life cognitive stimulation, as provided by higher levels of education, for brain health in late-life [16].

Further, accumulating evidence suggests that cardiovascular disease (CVD) risk factors such as hypertension, smoking, diabetes, and obesity play an instrumental role in neurodegenerative processes and contribute to increasing the risk of dementia (for a review see [36]). Notably, such CVD risk factors have been linked to the progression of MRI markers of brain ageing (e.g., white matter hyperintensities, brain atrophy, ventricular mass) [37–42] and cognitive decline (verbal memory, visuospatial memory, and executive function) [42–45]. Specifically, hypertension has been linked to late-life hippocampal atrophy and decreased neocortical grey matter volumes [46], as well as the appearance or progression of white lesions [42]. Smoking and type 2 diabetes [42, 47–49] have been linked to grey matter volume reductions in the medial temporal cortex, the precuneus, and posterior cingulate gyrus, areas implicated in Alzheimer's disease [39]. Finally, adiposity, commonly assessed using BMI, has also been linked to atrophy in the temporal cortex, frontal lobes, putamen, caudate, precuneus, thalamus, and white matter [40, 42, 50–52].

**Figure 1:**
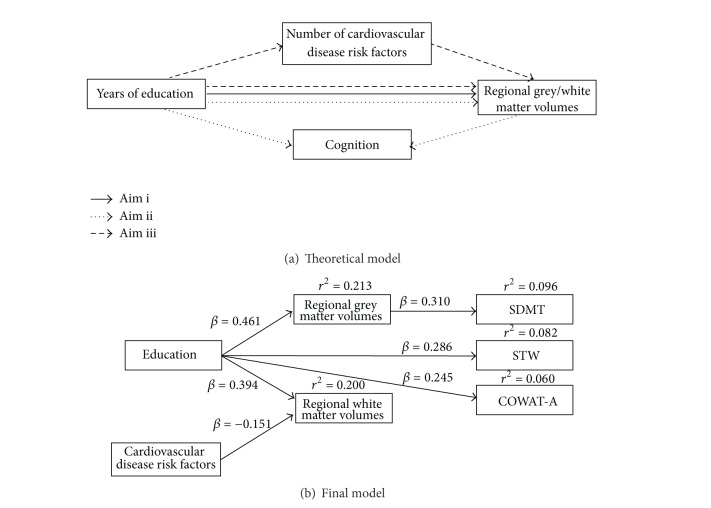
(a) Theoretical model testing: (1) the association between education and regional grey and white matter volumes (Aim i); (2) the mediating effects of regional grey/white matter volumes on the association between education and cognition (Aim ii); and (3) the mediating effects of number of cardiovascular disease risk factors on the association between education and regional grey/white matter volumes (Aim iii). (b) Significant bias corrected standardised regression weights from a bootstrap of 1000 samples. The final model depicts the significant associations between education, cardiovascular disease risk factors, regional brain volumes, and cognitive test performance.

While research has started to consider the direct effect of individual CVD risk factors on brain structure, little is known about how such factors modulate or contribute to the effect of education on brain structure and cognition. The aim of this study is therefore to examine the interplay between education, regional brain volumes, and cognitive function, while assessing the extent to which CVD risk factors mediate these associations. The theoretical model depicting the hypothesised relationships is displayed in Figure 1(a). This model proposes that (1) regional brain volumes mediate the relationship between education and cognitive function and (2) CVD risk factors mediate, at least in part, the relationship between education and regional brain volumes.

Using data from the PATH Through Life project [53], this study will therefore be guided by four main aims: (i) to establish the association between level of education and regional grey and white matter volumes; (ii) to identify bivariate associations between these regional brain volumes and cognitive functioning; (iii) to determine whether these identified regional grey/white matter volumes mediate the association between education and cognition; and (iv) to ascertain whether number of CVD risk factors mediate the association between education and regional grey/white matter volumes.

## 2. Material and Methods

### 2.1. Subjects

Subjects were sampled from the Personality and Total Health Through Life (PATH) project, a large longitudinal study of ageing aimed at investigating the course of mood disorders, cognition, health, and other individual characteristics across the lifespan [53]. PATH surveys 7485 individuals in three age groups of 20–24, 40–44, and 60–64 years at baseline. Follow-up is every four years over a period of 20 years. PATH surveys residents of the city of Canberra and the adjacent town of Queanbeyan, Australia, who were randomly recruited through the electoral roll [53]. Enrolment to vote is compulsory for Australian citizens, making this cohort representative of the population. The study was approved by the Australian National University Ethics Committee.

The present investigation is focused on the older participants (60s cohort). Of the 2551 randomly selected older PATH subjects included in the study at wave 1, 2076 consented to be contacted regarding an MRI scan. Of these, a randomly selected subsample of 622 subjects was offered an MRI scan. 478 (77%; 252 men) eventually completed MRI scanning. Of these, 360 subjects (198 men; age range 68–74) were rescanned at wave 3. Of those with MRI scans, ninety-four (26.1%) were excluded from the current analyses due to gross brain abnormalities (e.g., tumours, hydrocephalus; *n* = 18), a history of epilepsy, Parkinson's disease, or stroke (*n* = 30) or a clinical diagnosis of mild cognitive impairment or dementia (based on a full neuropsychological assessment using the criteria for mild cognitive impairment by Winblad [54] and the Diagnostic and Statistical Manual of Mental Disorders, 4th edition criteria for dementia; *n* = 46; see Anstey et al. [55] for detailed information on the diagnostic procedure).

**Figure 2:**
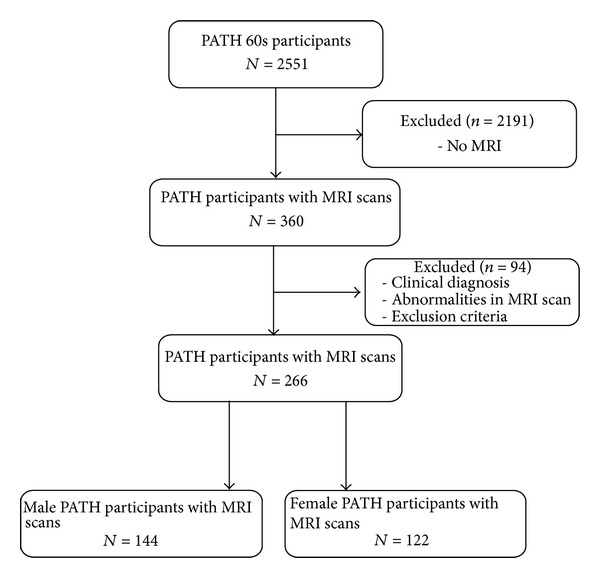
Sample inclusion.

Following all exclusions, the final sample included 266 cognitively healthy participants. Using this total sample, initial voxel-wise regression analyses with education as predictor and controlling for age and gender were performed. Sensitivity analyses identified a significant education-by-gender interaction. Subsequent analyses were therefore stratified by gender (male = 144; female = 122) (Figure 2). The final sample did not differ from the larger PATH sample on gender (*χ* ^2^ (1, *N* = 2551) = 0.748, *P* = 0.387) but had completed significantly more years of education (14.3 versus 13.7; *t* (2544) = −2.96, *P* < 0.01).

### 2.2. Sociodemographic and Health Measures

Sociodemographic information for race, alcohol consumption, smoking, hypertension, diabetes, and depression were assessed using self-report. Participants were asked about their current or past history of smoking, hypertension, and diabetes at each wave of the study. Questions include “Do you currently smoke?”; “Have you ever smoked regularly”; “Has your doctor told you that you suffer from high blood pressure?”; and “Have you been told by your doctor that you suffer from diabetes?”. Body mass index (BMI) was based on subjects' self-report of weight and height and computed using the formula weight (kg)/height(m)^2^. Overweight was classified as a BMI score of 25 or higher.

### 2.3. Years of Education and Cardiovascular Disease Risk Factors

Years of education was assessed at baseline and represented total years of education (range 5 to 19 years). Briefly, the highest level of education attained was computed based on a combination of responses to the following items: (i) the amount of primary (elementary) and secondary schooling; (ii) the highest level of postsecondary/tertiary education attained; (iii) number of years taken to complete postsecondary/tertiary education; (iv) present courses of study; (v) time taken on present courses of study; and (vi) whether present study is being completed on a full- or part-time basis.

A composite score for CVD risk factors (score range 0–4) was calculated based on self-reported (current or past) history of smoking, hypertension, and diabetes, as well as a BMI greater than 25 (i.e., cutoff for overweight). Specifically, CVD risk factors are defined as the total number of these risk factors that each participant screened positive for. Individuals not reporting any of these risk factors were scored 0. Individuals reporting only one of these risk factors (either smoking, hypertension, diabetes, or a BMI over 25) were scored as 1. Individuals reporting a combination of any two of these risk factors (either smoking, hypertension, diabetes, or a BMI over 25) were scored as 2. Individuals reporting any three of these risk factors (either smoking, hypertension, diabetes, or a BMI over 25) were scored as 3. Individuals reporting all four of these risk factors (smoking, hypertension, diabetes, and a BMI over 25) were scored as 4.

### 2.4. Neuropsychological Tests

Cognitive performance was assessed at wave 3. All participants completed all cognitive assessments as part of the standardised procedure for test administration (same order of test administration for each participant). To avoid possible interaction effects between cognitive assessments the neuropsychological tests were separated by physical measure assessments (e.g., measuring of blood pressure, waist circumference, or lung capacity).

Information processing speed were assessed using the Symbol-Digit Modalities Test (SDMT) [56]. Participants were given 90 seconds to complete this task. Episodic memory was assessed using the first trial of the California Verbal Learning Test for both immediate (IR) and delayed recall (DR) [57]. Participants were read words at one second intervals and there was approximately one minute between immediate and delayed recall tests. Participants were not limited in the amount of time they could take to recall at either trial. Executive function of verbal fluency was assessed using the Controlled Oral Word Association Test (COWAT) for both A- and F-words. Participants were timed for 60 seconds to list as many words starting with the letters A and F, respectively. Verbal intelligence (lexical decision making ability) was assessed using the Spot-the-Word Test (STW) [58]. Participants were asked to decide between a real word and a nonsense word invented to look like a real word but has no meaning (e.g., to decide between the words: “bread” and “glot”). The Boston Naming Test (BNT) [59] was used to assess language function. Participants were shown pictures of objects and asked to name the object. Verbal working memory was assessed through the Digit-Backwards Span test (DB), a subtest of the Wechsler memory scale [60]. In the DB test, participants were read numbers at one-second intervals. When participants incorrectly repeated both trials no further trials were given. Attention and executive function of task switching were assessed using the Trail Making Test (TMT) parts A and B [61]. For both tests (TMT-A and TMT-B) completion times and number of errors were recorded. TMT-A and TMT-B were discontinued if 5 errors were made or the completion time exceeded 300 seconds.

### 2.5. Data Acquisition

MRI scans used in this study were taken at wave 3. Subjects were scanned on a Siemens 1.5 T Avanto scanner (Siemens Medical Solutions) for T1-weighted three-dimensional structural MRI. The T1 weighted MRI was acquired in sagittal orientation using the following parameters: repetition time (TR)/echo time (TE) = 1.16/~0.8 ms; flip angle = 15°; matrix size = 512 × 512; and slice thickness = 1.0 mm, resulting in a final voxel size of 1 × 0.5 × 0.5 mm.

### 2.6. Image Processing

All images were preprocessed using the MINC imaging toolbox (MINC; http://en.wikibooks.org/wiki/MINC). Images went through automatic QC to identify outliers via image histogram clamping and comparisons to the group minimum deformation average [62]. Images were then B0 MRI inhomogeneity corrected using N3 [63] and normalised via a linear correction to a global intensity model [62].

Optimized voxel-based morphometry (VBM) analyses were conducted using Statistical Parametric Mapping 8 (SPM8; Wellcome Department of Cognitive Neurology, London, UK, 2003) on Matlab 7.12 (Math Works, Natick, MA, USA, 2002). Images were first segmented into grey matter, white matter, and cerebrospinal fluid [64]. Grey and white matter segmentations were further normalised to the sample template (population representative) which was generated by the diffeomorphic anatomical registration through exponentiated lie algebra (DARTEL) algorithm from participants' complete images [65]. DARTEL is a nonlinear warping technique that minimises structural variation between subjects and has been evidenced to be more accurate than the standard normalisation approach in SPM [66, 67]. Briefly, segmented images were registered, normalised, and modulated to fit the DARTEL space, creating a DARTEL template based on the deformation fields produced in the segmentation procedure in which all individual deformation fields are warped (and modulated) to match this template [68]. Images were smoothed using a 8 mm, full-width-at-half-maximum Gaussian kernel to increase the signal-to-noise ratio, with each voxel of the resulting grey and white matter images representing the absolute amount of grey and white matter volume equivalent to their volume per unit before normalisation [68].

### 2.7. Image Data Analyses

Absolute total grey and white matter volumes were calculated using the native space grey and white matter segmentations. Smoothed grey and white matter density images were used in the voxel-wise regression analyses with total years of education as predictor. To account for the effects of possible confounding factors, age was controlled for. Results were assessed at a family-wise corrected level of *α* = 0.05. To avoid false positives resulting from noise only, clusters >20 voxels were considered significant.

### 2.8. Statistical Analyses

Demographic characteristic analyses were conducted using IBM SPSS Statistics 21.0. All analyses were stratified by gender. To explore our aims we first used VBM to identify associations between education with regional grey and white matter densities. Grey and white matter densities at significant voxels were extracted at the cluster level using the SPM8 “eigenvariate” extraction tool and standardised to *Z*-scores. To identify significant associations between the variables of interest (i.e., education, CVD risk factors, regional grey/white matter volumes, and cognition) bivariate Spearman's Rank Order correlation analyses were performed. Significant bivariate associations provided the framework for inclusion in subsequent analyses. To explore possible mediating effects of regional grey/white matter volumes and CVD risk factors on the association between education and cognition, a path analysis was utilised within a structural equation modelling (SEM) framework using IBM AMOS version 21. Benefits to using an SEM framework include exploring multiple mediation and multiple outcome variables in a single model, the capacity to utilise alternative estimation methods such as maximum likelihood (MLE), or asymptotically distribution-free (ADF) estimation and the ease of producing robust standard errors or bias corrected confidence from bootstrapping techniques. To adjust for non-Gaussian scale distributions and likely violations of multivariate normality, we estimated our SEM with ADF estimation and undertook a bootstrap of *n* = 1000 samples to generate robust standard errors. Missing data for cognitive measures were imputed using the EM algorithm in SPSS.

## 3. Results

Participants' demographic characteristics are presented in Table 1.

### 3.1. Associations between Education and Regional Brain Volumes

**Figure 3:**
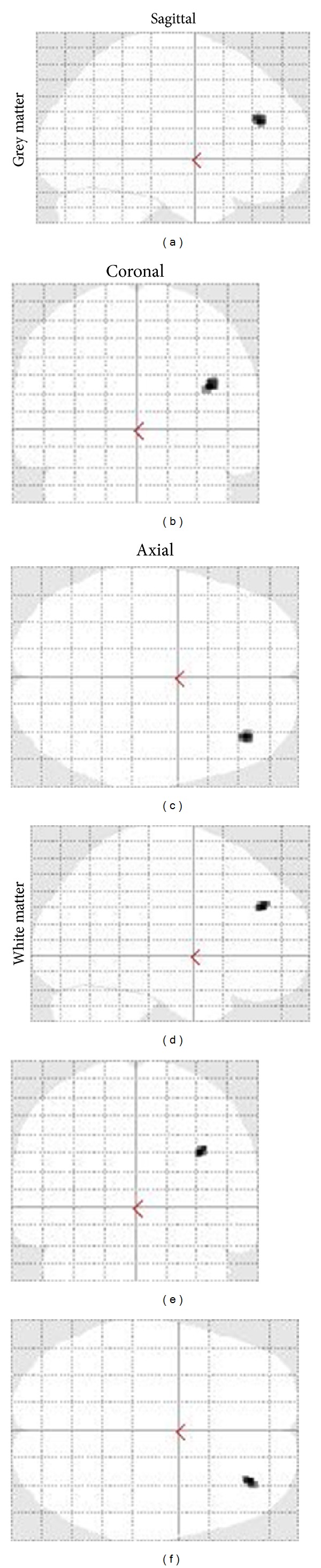
Sagittal, coronal, and axial representation of larger regional grey and white matter associated with education in men (*P* < 0.05, FWE).

Using family-wise corrected VBM analyses (*P* < 0.05, FWE) adjusted for age, higher education was associated with larger regional grey matter volumes of the* Right Middle Frontal Gyrus*; (*Region 1*) and larger regional white matter volumes of the* Right Middle Frontal Gyrus* (*Region 2*) in men (Table 2 and Figure 3). No significant family-wise corrected associations between education and regional volumes were reported in women. Consequently further analyses focused only on men.

### 3.2. Bivariate Relationships

Bivariate Spearman's Rank Order correlations between education, CVD risk factors, regional grey/white matter volumes, and cognitive function in men are reported in Table 3. A number of bivariate associations were found. Regional* grey matter volumes* of the Right Middle Frontal Gyrus (Region 1) were associated with better performance on the cognitive measures of IR (*r*_*s*_ = 0.173, *P* = 0.038), SDMT (*r*_*s*_ = 0.267, *P* = 0.001), COWAT-A words (*r*_*s*_ = 0.188, *P* = 0.024), TMT-A (*r*_*s*_ = −0.183, *P* = 0.028), and STW (*r*_*s*_ = 0.175, *P* = 0.035). Overall it would appear that only the association with SDMT reported a substantive association, with the others only just reaching statistical significance. No significant bivariate correlations were found between regional* white matter volumes* of the Right Middle Frontal Gyrus (Region 2) and cognitive test performance.

*Level of education* was positively associated with grey matter volumes of Region 1 (*r*_*s*_ = 0.424, *P* = 0.000), white matter volumes of the Region 2 (*r*_*s*_ = 0.437, *P* = 0.000), and CVD risk factors (*r*_*s*_ = 0.185, *P* = 0.026). Level of education was also associated with better performance on the cognitive assessments of SDMT (*r*_*s*_ = 0.240, *P* = 0.004), COWAT-A (*r*_*s*_ = 0.240, *P* = 0.004), TMT-A (*r*_*s*_ = −0.197, *P* = 0.018), and STW (*r*_*s*_ = 0.331, *P* = 0.000).

Finally, a higher number of* CVD risk factors* were associated with smaller regional white matter volumes of Region 2 (*r*_*s*_ = −0.235, *P* = 0.005) and poorer performance on the TMT-A (*r*_*s*_ = 0.174, *P* = 0.037). No significant bivariate associations were demonstrated between CVD risk factors and regional grey matter volumes (*r*_*s*_ = −0.108, *P* = 0.196;* n.s.*). Overall, these findings justified the exploration of the associations between education, CVRF, regional brain volumes, and cognitive function in an SEM model in order to examine possible mediation mechanisms.

### 3.3. Modelling the Education-Volume-Cognition Relationships

Following our theoretical baseline model (see Figure 1(a)), we examined the possible mediating role of regional grey/white matter volumes on the relationship between CVD risk factors, education, and cognitive functioning. All cognitive functioning indicators were regressed onto education, except for IR which did not have a statistically significant bivariate association with education (*P* > 0.05). Following from the results of the VBM analyses, extracted regional grey (Region 1) and white matter (Region 2) volumes were also regressed onto education, whilst only the white matter region regressed on CVD risk factors since grey matter reported no significant bivariate association. Those bivariate associations with CVD risk factors which reached statistical significance were also included in the SEM (e.g., TMT-A: *r*_*s*_ = 0.174, *P* = 0.037; regional white matter volumes: *r*_*s*_ = −0.235, *P* = 0.005). Overall, results indicated that data fit our baseline model well (*χ* ^2^ (*df* = 11, *N* = 144) = 4.578; *P* = 0.950, RMSEA = 0.000 (90% CI = 0.000–0.002), and BIC = 173.552; AGFI = 0.981).

However, not all parameters were statistically significant in the SEM. Of particular note, TMT-A was now not associated with either regional grey matter volumes (*β* = −1.266 (95% CI = −.3.011–0.380), *P* = 0.209), education (*β* = −0.360 (95% CI = −1.098–0.330), *P* = 0.358), or CVD risk factors (*β* = 1.286 (95% CI = −0.050–2.987), *P* = 0.105). Examination of the covariances and correlations between the dependent variables indicated a substantial correlation between SDMT and TMT-A (*r* = −0.505,  *P* < 0.001). Although multicollinearity is typically problematic where *r* > 0.7, a partial correlation analysis removing the effect of SDMT from the associations among TMT-A, regional grey matter volume, and education rendered TMT-A nonsignificant. Therefore, this latter variable was excluded from further analyses and SDMT retained in the model.

Also nonsignificant associations were reported between regional grey matter volumes and both COWAT-A (*β* = 0.731, (95% CI = −0.294–1.505), *P* = 0.245) and STW (*β* = 0.201 (95% CI = −0.343–0.899), *P* = 0.498) when adjusted for education. Based on the size of the previously reported bivariate correlations and associated significance values, it is not surprising that these effects now failed to reach statistical significance in the adjusted SEM model. Importantly, the association between education and SDMT (*β* = 0.460 (95% CI = −0.078–1.074), *P* = 0.156) was now also nonsignificant in the SEM model, suggesting that regional grey matter volumes fully mediated the effect of education on SDMT.

Due to the large number of redundant parameters in the baseline model, a second adapted and reduced model was therefore reestimated excluding these nonsignificant parameters. Standardised effects are reported in Figure 1(b). Bias-corrected unstandardised estimates and 95% CI from a bootstrap of 1000 samples are reported in Table 4,* Reduced Model*. Although the reduced model reported some decline in model fit for some indices (*χ* ^2^ (*df* = 16, *N* = 144) = 17.316, *P* = 0.365; AGFI = 0.951, RMSEA = 0.024 (90% CI = 0.000–0.083)) in comparison with the baseline model, these indices are still within acceptable norms to reflect good model fit and the BIC (BIC = 116.713) even reported an improvement of fit to the data.

A number of important findings are worth highlighting. The bootstrap analysis indicated significant positive associations between education and the regional grey/white matter volumes, STW, and COWAT-A (see Table 4). Also, a substantive association was reported between regional grey matter volumes and SDMT. Education had direct effects on COWAT-A and STW but regional grey and white matter volumes were not associated with COWAT-A or STW. Therefore, no indirect effects for education were reported for these cognitive indicators. Despite a significant negative bivariate correlation, the association between education and CVD risk factors was now no longer statistically significant. Similarly, the association between regional grey matter volumes and IR was also now no longer significant. Whilst there is little support for an overall mediation role of regional volumes on the association between education and cognitive function, the SEM indicated one strong full mediation effect of regional grey matter. The direct effect for education on SDMT was fully mediated by regional grey matter, with approximately 46% of the association between regional grey matter volumes and SDMT being attributable to education.

### 3.4. Sensitivity Analyses

While age was not included in the main model due to the study design using a narrow age cohort (age range 68–73 years), supplementary SEM analyses were performed controlling for those bivariate associations with age which reached statistical significance (e.g., SDMT: *r*_*s*_ = −0.169, *P* = 0.043; STW: *r*_*s*_ = 0.214, *P* = 0.010). These analyses produced essentially the same results, including a good model fit (*χ* ^2^ (*df* = 18, *N* = 144) = 20.376; *P* = 0.312, RMSEA = 0.030 (90% CI = 0.000−0.083), BIC = 204.260; AGFI = 0.986) and no association between TMT-A with either regional grey matter volumes (*β* = −1.124 (95% CI = −3.938–0.832), *P* = 0.203), education (*β* = −0.379 (95% CI = −1.405–0.595), *P* = 0.463), or CVD risk factors (*β* = 1.456 (95% CI = −0.344–3.735), *P* = 0.100). Additionally, no significant associations were reported between SDMT and age (*β* = −0.706 (95% CI = −1.513–0.068), *P* = 0.079) or STW and age (*β* = −0.393 (95% CI = −0.173–0.869), *P* = 0.142), indicating their redundancy in the current model.

## 4. Discussion

This study investigated the extent to which (1) associations between education and cognitive function are mediated by regional brain volumes and (2) the extent to which associations between education and regional brain volumes are mediated by CVD risk factors. Three important findings were made: (1) higher educational attainment was associated with larger regional grey and white matter volumes, but in men only; (2) larger regional grey matter volumes mediated the association between education and cognition but only in relation to information processing speed; and (3) the number of CVD risk factors did not mediate the association between education and regional volumes. The following sections will discuss the implications of these findings.

### 4.1. Regional Volumes: An Index of Cognitive/Neural Reserve

The current study found higher educational attainment to be associated with larger regional grey and white matter volumes in the prefrontal cortex, specifically in the right middle frontal gyrus. These findings are in line with findings from animal studies, which have shown different experiences to affect brain structure, including vasculature, number of cells and synapses, and brain cell connections in adult life [69, 70], thus contributing to a “reserve” which may buffer against neuropathological effects by providing structural and functional compensation. Stern et al. [71] refers to this as “neural reserve,” where modulating factors such as life events (e.g., educational or occupational experience, leisure activity) contribute to more efficient brain networks or networks which have greater capacity when faced with increased demand. Interestingly in this study, higher educational attainment was specifically associated with larger grey and white matter regional volumes in the middle frontal gyrus, an area associated with volume reductions and functional changes in healthy ageing [72, 73]. These findings therefore provide additional evidence in support of a protective effect of education by contributing, not only to a cognitive reserve, but also to a neural reserve, which may provide compensation for ageing and pathological processes.

### 4.2. Regional Volumes: A Mediator

Importantly, while this study identified regional grey and white matter volumes, these regional volumes were not associated with cognitive function, except for the association between regional grey matter volumes and SDMT performance (i.e., larger regional grey matter volumes in the middle frontal gyrus are associated with better SDMT performance). Notably, divided attention, visual scanning, visual tracking, perceptual speed, motor speed, and memory all contribute to SDMT performance [74]. Previous anatomical studies have linked regional grey and white matter volumes in the prefrontal cortex with working memory performance [75] and more specifically the right middle frontal gyrus with spatial working memory [76]. This association may thus provide a possible explanation for the observed mediation of regional grey matter volumes on the association between education and performance on the SDMT in the current study.

### 4.3. Cardiovascular Disease Risk Factors: Not a Mediator

Finally, a higher number of CVD risk factors were only associated with smaller regional white matter volumes and did not mediate the association between education and regional white matter volumes. These findings are interesting as they suggest that exposure to a higher number of CVD risk factors may increase the risk of white matter pathology, irrespective of the protective effect provided by higher educational attainment and its associated cognitive and neural reserve. These findings are in accordance with the literature linking these individual vascular factors to cerebral ageing and cognitive decline (e.g., [42]) but extend them to consider the comorbid effect of multiple CVD risk factors on cerebral structure and cognition. Further, while these findings extend previous research linking individual CVD risk factors to more severe AD pathology, when compared to individuals without risk factors (e.g., [77, 78]), future research is needed to improve understanding of the impact that comorbid CVD risk factors have on white matter structures and the protective effect associated with higher educational attainment, especially in a longitudinal framework.

### 4.4. Limitations and Strengths

The limitations and strengths of this study must also be considered. First, while this study has a cross-sectional design, there is a strong theoretical argument why higher education may impact cerebral structures, cognitive performance, and CVD risk factors. However, longitudinal studies which consider other possible pathways and reciprocal effects between education, cerebral structure, cognition, and CVD risk factors, as well as the possible role of other factors, are needed. Further, the use of a cross-sectional design may have limited our ability to detect a mediating role of CVD risk factors in the associations between education, cerebral structures, and cognition, as it is possible that effects may only become evident at an older age. Longitudinal studies are therefore needed in which the role of CVD risk factors in the associations between education, cerebral structures, and cognition is further investigated. Second, while PATH is a population representative study in which participants were randomly selected into the neuroimaging substudy from the larger population-representative cohort, it may not be completely representative of the population at large. Further, the selection procedure which excluded those with neurocognitive disorders and brain abnormalities as well as the use of a narrow age cohort also decreases the representativeness of the sample, making it more difficult to generalise the current findings to other age groups. Nonetheless, the overall effect of this methodology was to select overall healthier individuals and therefore any effect detected in this study is likely to be an underestimate of those applying to the general population. Third, it is well known that SDMT performance is sensitive to level of education [79]. This may have biased the current findings, as regional volumes were identified based on their association with education in the VBM analyses. Fourth, the summation of CVD risk factors may be a somewhat simplistic approach, limiting the range and attributing equal importance to all four variables. Future research is needed in which more sensitive measures are used to better reflect the complex interplay between individual CVD risk factors.

Despite these limitations, this study is characterised by significant strengths. Study strengths include the large sample size and a population-based sampling frame. Further, to ensure the effects detected related to educational attainment, individuals with other neurological disorders were excluded. This allowed for the investigation of long term associations between level of education, brain structure, CVD risk factors, and cognitive performance despite a cross-sectional design.

### 4.5. Conclusion

In conclusion, the current findings provide further evidence of the protective effect of education for brain health and cognition. However, it also highlighted the importance of considering the possible interactive effects of comorbid CVD risk factors in increasing the risk of white matter pathology, irrespective of the protective effect provided by education-related cognitive and neural reserve.

## Figures and Tables

**Table 1:** Sample descriptive.

Characteristics	Overall Sample (*N* = 266)	Male (*N* = 144)	Female (*N* = 122)	*F*/*χ* ^2^ male versus female
Age, years (SD)	70.4 (1.42)	70.4 (1.44)	70.4 (1.40)	0.003
Range	68–73	68–73	68–73	
Race				1.74
Caucasian, *N* (%)	254 (95.5)	137 (95.1)	117 (95.9)	
Asian, *N* (%)	6 (2.3)	4 (2.78)	2 (1.64)	
Other, *N* (%)	4 (1.5)	1 (0.694)	3 (2.46)	
Education, years (SD)	14.2 (2.57)	15.0 (2.27)	13.3 (2.61)	32.3∗∗∗
Range	5–19	9–19	5–19	
Cardiovascular disease risk factors				7.41
None, *N* (%)	15 (5.6)	7 (4.9)	8 (6.6)	
One, *N* (%)	70 (26.3)	36 (25.0)	34 (27.9)	
Two, *N* (%)	102 (38.3)	49 (34.0)	53 (43.4)	
Three, *N* (%)	62 (23.3)	39 (27.1)	23 (18.9)	
Four, *N* (%)	17 (6.4)	13 (27.1)	4 (3.3)	
MMSE, score (SD)	29.4 (0.874)	29.3 (0.881)	29.4 (0.862)	2.13
Range	26–30	26–30	26–30	
BMI, score (SD)	26.6 (4.91)	26.5 (3.73)	26.8 (6.02)	0.141
Range	18–60	20–39	18–60	
Mean arterial blood pressure, level (SD)	103.9 (11.2)	105.0 (11.5)	102.6 (10.7)	3.02
Range	81–155	82–155	81–130	
Average systolic pressure, level (SD)	149.7 (19.5)	150.3 (19.9)	148.9 (18.9)	0.334
Range	108.5–219.0	108.5–219.0	109–205	
Average diastolic pressure, level (SD)	81.0 (9.84)	82.4 (10.0)	79.5 (99.41)	5.75∗
Range	57.5–124	59.5–124	57.5–104.5	
Smoke				4.25∗
History or current, *N* (%)	114 (42.9)	70 (48.6)	44 (36.1)	
Cognitive test performance				
Immediate recall, mean (SD)	6.89 (2.03)	6.47 (1.74)	7.40 (2.24)	14.7∗∗∗
Range	2–13	3–11	2–13	
Delayed recall, mean (SD)	6.07 (2.18)	5.70 (1.90)	6.50 (2.41)	9.13∗∗
Range	0–12	1–11	0–12	
Digit backward, mean (SD)	5.28 (2.03)	5.62 (2.01)	4.88 (1.99)	9.07∗∗
Range	1–10	1–10	1–10	
Symbol Digit Modalities Test, mean (SD)	49.7 (8.53)	49.4 (8.32)	49.9 (8.79)	0.32
Range	16–75	20–75	16–71	
COWAT A-words, mean (SD)	12.8 (5.39)	13.3 (5.39)	12.3 (5.38)	2.49
Range	0–30	2–30	0–29	
COWAT F-words, mean (SD)	14.3 (4.94)	14.7 (4.98)	13.9 (4.88)	1.59
Range	4–31	6–31	4–30	
Spot the Word, mean (SD)	53.5 (5.08)	54.4 (4.55)	52.4 (5.46)	10.2∗∗
Range	34–60	34–60	37–60	
Boston Naming Test, mean (SD)	13.9 (1.31)	13.9 (1.29)	13.8 (1.34)	1.51
Range	7–15	7–15	9–15	

*P < 0.05;  **P < 0.01;  ***P < 0.001.

**Table 2:** Education-related atlas coordinates, cluster extents, *P* and *T* values, and regional descriptions in men.

	MNI coordinates (*x*, *y*, *z*)	Cluster extent (*k*)	Cluster-level *P* corrected	*T*	Region description (for cluster peak)
Grey matter					
Region 1	39, 39, 22	37.8	FWE *P* < 0.05	5.65	Right middle frontal gyrus
White matter					
Region 2	33, 41, 28	29	FWE *P* < 0.05	5.36	Right middle frontal gyrus

**Table 3:** Bivariate associations between education, cardiovascular risk factors, regional brain volumes, and cognitive test performance.

Cognitive Tests														
	GM	WM	Education	CVD risk factors	IR	DR	DB	SDMT	STW	COWAT A	COWAT F	TMT-A	TMT-B	BNT
Male														
Grey matter														
Region 1	—	0.516∗∗∗	0.424∗∗∗	−0.108	0.173∗	0.095	0.093	0.267∗∗	0.175∗	0.188∗	0.151	−0.183∗	−0.051	0.085
White matter														
Region 2	—	—	0.437∗∗∗	−0.235∗∗	0.153	0.086	−0.012	0.095	0.112	0.148	0.105	−0.157	−0.062	0.067
Education	—	—	—	−0.185∗	0.124	0.101	0.187∗	0.240∗∗	0.331∗∗	0.240∗∗	0.273∗∗∗	−0.197∗	−0.133	0.033
CVRF	—	—	—	—	−0.033	0.005	−0.082	−0.095	−0.047	−0.051	−0.079	0.174∗	0.129	0.064
Age	−0.042	−0.103	0.000	−0.056	0.115	0.072	−0.073	−0.169∗	0.214∗	0.163	0.001	0.076	0.199∗	0.030
Female			No correlations between regional volumes, education, and cognition were performed in women											

GM: grey matter regional volumes, WM: white matter regional volumes, CVD risk factors: cardiovascular disease risk factors, IR: California Verbal Learning test for immediate recall, DR: California verbal learning test for delayed recall, DB: digit span backwards from the Wechsler Memory Scale, SDMT: Symbol-Digit Modalities Test, STW: Spot the Word Test, COWAT-A: Controlled Oral Word Association Test for A-words, COWAT-F: Controlled Oral Word Association Test for F-words, TMT-A: Trail Making Test Part A, TMT-B: Trail Making Test Part B, and BNT: Boston Naming Test.

***P < 0.001; **P < 0.01; *P < 0.05; —: no effect to report.

**Table 4:** Unstandardised biased corrected effects with 95% CI and significance level from a bootstrap of 1000 samples.

	CVD risk factors	Grey matter volumes	White matter volumes	IR	SDMT	COWAT-A	STW
	*β* (95% CI)	*β* (95% CI)	*β* (95% CI)	*β* (95% CI)	*β* (95% CI)	*β* (95% CI)	*β* (95% CI)
Reduced model							
Education	−0.085 (−0.174–0.009)	0.204∗∗ (0.100–0.297)	0.176∗∗∗ (0.108–0.247)	—	—	0.555∗ (0.113–1.032)	0.571∗∗∗ (0.272–0.964)
CVRF	—	—	−0.143∗ (−0.291–−0.001)	—	—	—	—
Grey matter volumes	—	—	—	0.268 (−0.051–0.595)	2.542∗∗ (1.065–4.218)	—	—
White matter volumes	—	—	—	—	—	—	—

CVD risk factors: cardiovascular disease risk factors, IR: immediate recall, SDMT: Symbol-Digit Modalities Test, COWAT-A: Controlled Oral Word Association Test for A-words, STW: Spot the Word, and CI: confidence interval.

*P < 0.05; **P < 0.01***P < 0.001; —: no effect to report.
